# Triple-Site Ectopic Thyroid Involving the Submandibular, Lingual, and Infrahyoid Regions: A Rare Case

**DOI:** 10.7759/cureus.94059

**Published:** 2025-10-07

**Authors:** Varun Tej, Satyanarayana Kummari, Abhishek J Arora, Annapurna Srirambhatla, Syed Ashfaq

**Affiliations:** 1 Radiodiagnosis, All India Institute of Medical Sciences, Bibinagar, Hyderabad, IND

**Keywords:** 99mtc thyroid scintigraphy, colloid nodule, ectopic thyroid gland, lateral thyroid, lingual thyroid, overt hypothyroidism, submandibular region, submandibular thyroid, ultrasound neck

## Abstract

Thyroid gland development normally begins in utero during the fourth week of pregnancy. It advances to its definitive anatomical location, situated between the second and fifth tracheal cartilages, often by seven weeks of gestation. An ectopic thyroid gland is characterized by its unusual location, which results from abnormal embryonic development. A 34-year-old female patient presented to the ENT department, primarily complaining of painless swelling in the right submandibular region that had been present for nine months. This swelling was insidious in onset and gradually progressive, with a notable increase in size occurring over the last three months. The patient has a documented history of hypothyroidism and is now undergoing treatment. The results of thyroid function testing indicated that the level of thyroid-stimulating hormone was 6.107 µIU/mL, total triiodothyronine was 1.269 ng/mL, and total thyroxine was 11.197 µg/mL. Radiological imaging modalities, such as ultrasound, computed tomography (CT), and magnetic resonance imaging (MRI) scans of the neck, showed a complex, thick-walled cystic lesion adjacent to the right submandibular gland, measuring 3.9 x 3.0 cm, with thick internal septa and another well-defined solid lesion in the right infrahyoid region. The CT and MRI scans revealed another round solid lesion at the base of the tongue. Normal thyroid gland tissue was not seen at its native place. Scintigraphy was not performed due to a recent use of an iodinated contrast medium for contrast-enhanced CT. The patient underwent ultrasound-guided fine-needle aspiration cytology (FNAC) for the right submandibular lesion; however, the cytology was non-diagnostic due to the predominant cystic component and rich vascularity. Because of suspicion of malignancy, non-diagnostic cytology, and a recent increase in the size of the swelling, the submandibular and infrahyoid lesions were completely surgically resected. Histopathology revealed cystic colloid tissue with benign thyroid cells displaying oncocytic changes and normal thyroid tissue in the submandibular and infrahyoid lesions. The patient was ultimately diagnosed with multifocal benign ectopic thyroid tissue without malignant transformation. The patient was instructed to continue with the medication for hypothyroidism.

The purpose of this case report is to present a very rare case of triple-site ectopic thyroid involving the submandibular, lingual, and infrahyoid regions, emphasizing the value of considering ectopic thyroid as a differential diagnosis in submandibular, infrahyoid, and lingual masses, particularly in the absence of an orthotopic gland.

## Introduction

Thyroid gland development normally begins in utero during the fourth week of pregnancy. From an embryological perspective, the thyroid gland is formed from two lateral cell clusters and a median cell cluster. It advances to its definitive anatomical location, situated between the second and fifth tracheal cartilages, often by seven weeks of gestation [1]. An ectopic thyroid gland is characterized by its presence in an unusual location, which results from abnormal embryonic development. Ectopic thyroid glands are predominantly located along the midline along their descent course; however, they may occasionally be detected off-midline [2].

The thyroid parenchyma is mostly produced by the median section, although the lateral portions make up 1-30% of the overall weight of the thyroid gland [2]. A lingual thyroid is frequently the result of failure in the median descent. A lateral ectopic thyroid gland may result from the failure to merge the lateral cell clusters with the median cluster [3]. Ectopic thyroid tissue is found in around one in every 100,000 to 300,000 people. The ratio of females to males is 4:1 [4]. We report an uncommon case of a middle-aged woman with a triple-site ectopic thyroid involving the submandibular, lingual, and infrahyoid regions, with colloid nodular changes in the submandibular and infrahyoid regions.

## Case presentation

A 34-year-old female patient presented to the ENT department, primarily complaining of painless swelling in the right submandibular region that had been present for nine months. This swelling was insidious in onset and gradually progressive, with a notable increase in size occurring over the last three months. There was no increase in the size of the swelling with food intake or talking. The patient has a documented history of hypothyroidism and is now undergoing treatment. Neck examination showed a well-defined, soft, solitary swelling in the right submandibular region, with mild tenderness and no palpable cervical lymph nodes. The swelling did not change upon deglutition or tongue protrusion. The ear, nose, oral cavity, and other head and neck examinations revealed no significant abnormality. Lab investigations were performed for thyroid status. The results of thyroid function testing (TFT) showed that the thyroid-stimulating hormone (TSH) level was 6.107 µIU/ml, total triiodothyronine (T3) level was 1.269 ng/mL, and total thyroxine (T4) level was 11.197 µg/mL.

**Figure 1:**
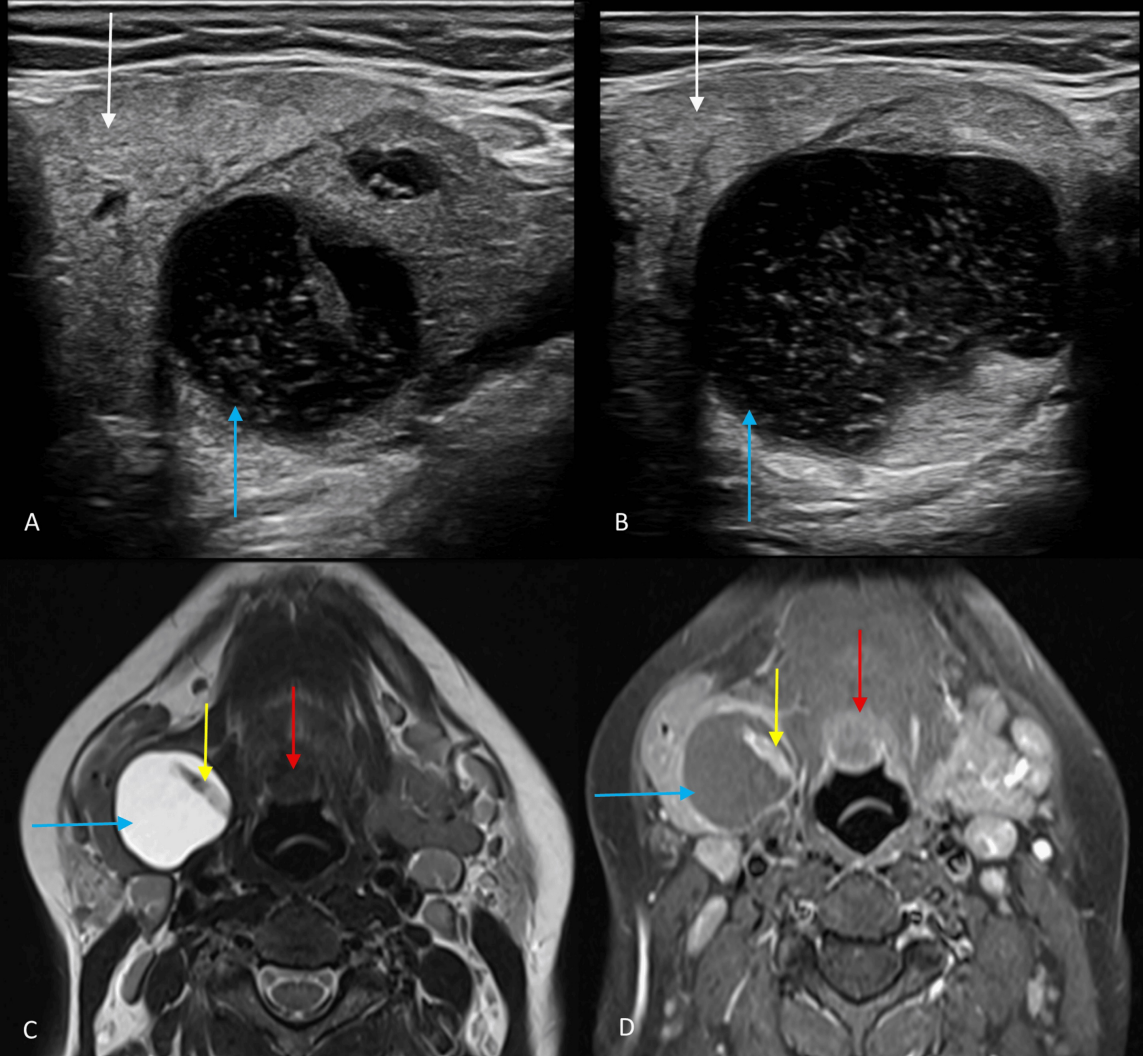
Ultrasound and MRI images of the submandibular region. (A and B) Gray scale ultrasound images of the right submandibular region show a well-defined predominantly cystic lesion with multiple internal echoes, eccentric solid components, and thick internal septation (blue arrow). The lesion is seen separate from the submandibular gland (white arrow). (C and D) Axial T2-weighted and post-contrast T1-weighted MRI of the neck show a well-defined round-oval-shaped cystic lesion in the right submandibular region, appearing hyperintense on T2-weighted images (blue arrow) with hypointense thick septation. The eccentric solid component showed enhancement on post-contrast T1-weighted images (yellow arrow). Enhancing lingual thyroid is also seen in these images (red arrow). MRI, magnetic resonance imaging

**Figure 2:**
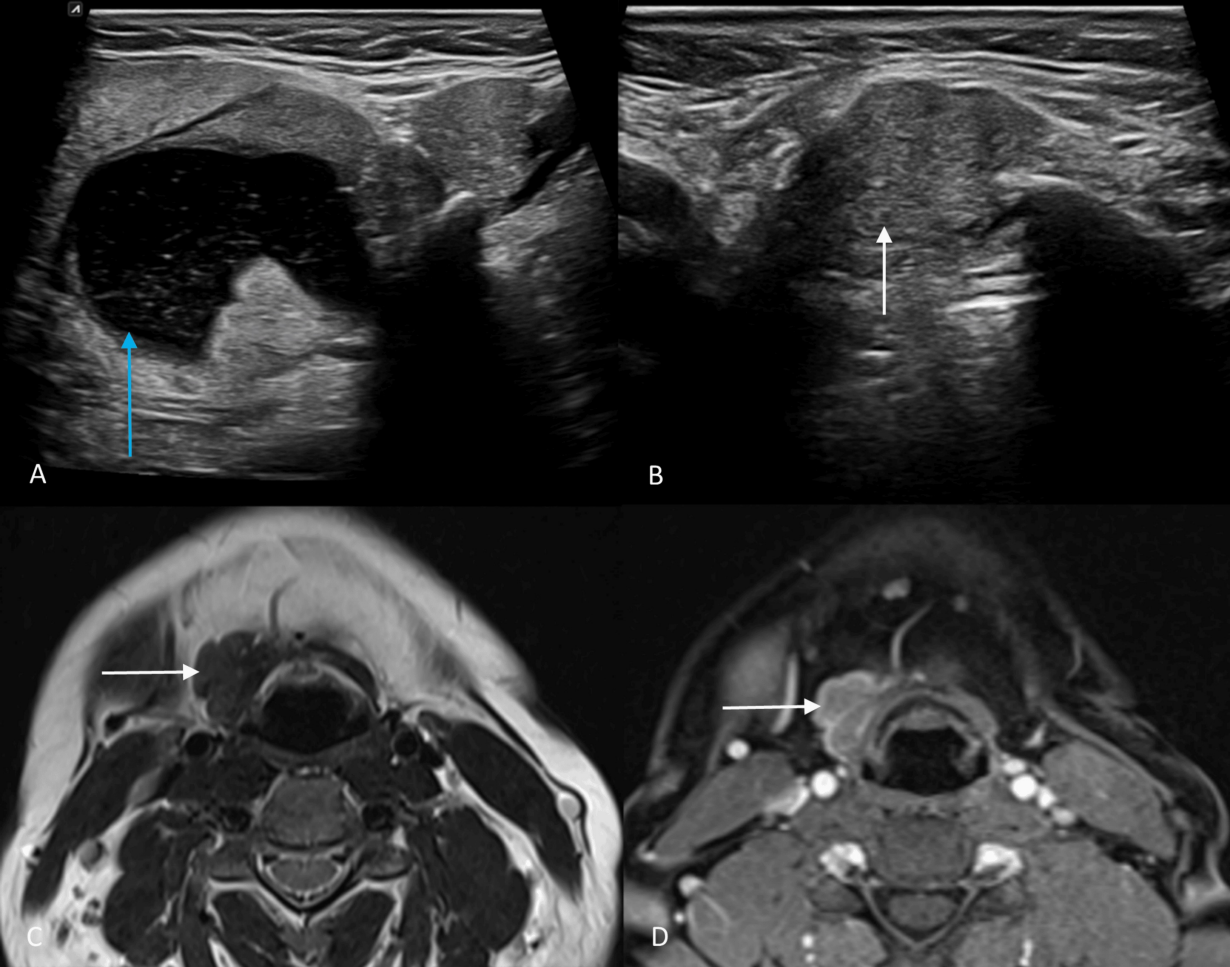
Ultrasound and MRI images of the suprahyoid and infrahyoid regions. (A and B) Gray-scale ultrasound images of the right suprahyoid and infrahyoid regions show both right submandibular cystic lesion (blue arrow) and infrahyoid solid lesion (white arrow). (C and D) Axial T2-weighted and post-contrast T1-weighted MRI of the neck shows a well-defined irregular solid lesion in the right infrahyoid region, appearing isointense on T2-weighted imaging (white arrow). It shows heterogeneous enhancement on post-contrast T1-weighted images (white arrow). MRI, magnetic resonance imaging

Ultrasound of the neck showed a complex anechoic thick-walled cystic structure adjacent to the right submandibular gland, measuring 3.9 x 3.0 cm, with multiple floating internal echoes, thick internal septa, and an eccentric hyperechoic solid component showing internal vascularity. Another well-defined hyperechoic lesion was found in the infrahyoid region at the right paramedian location, showing significant internal vascularity with ill-defined deeper margins with strap muscles (Figures 1, 2).

**Figure 3:**
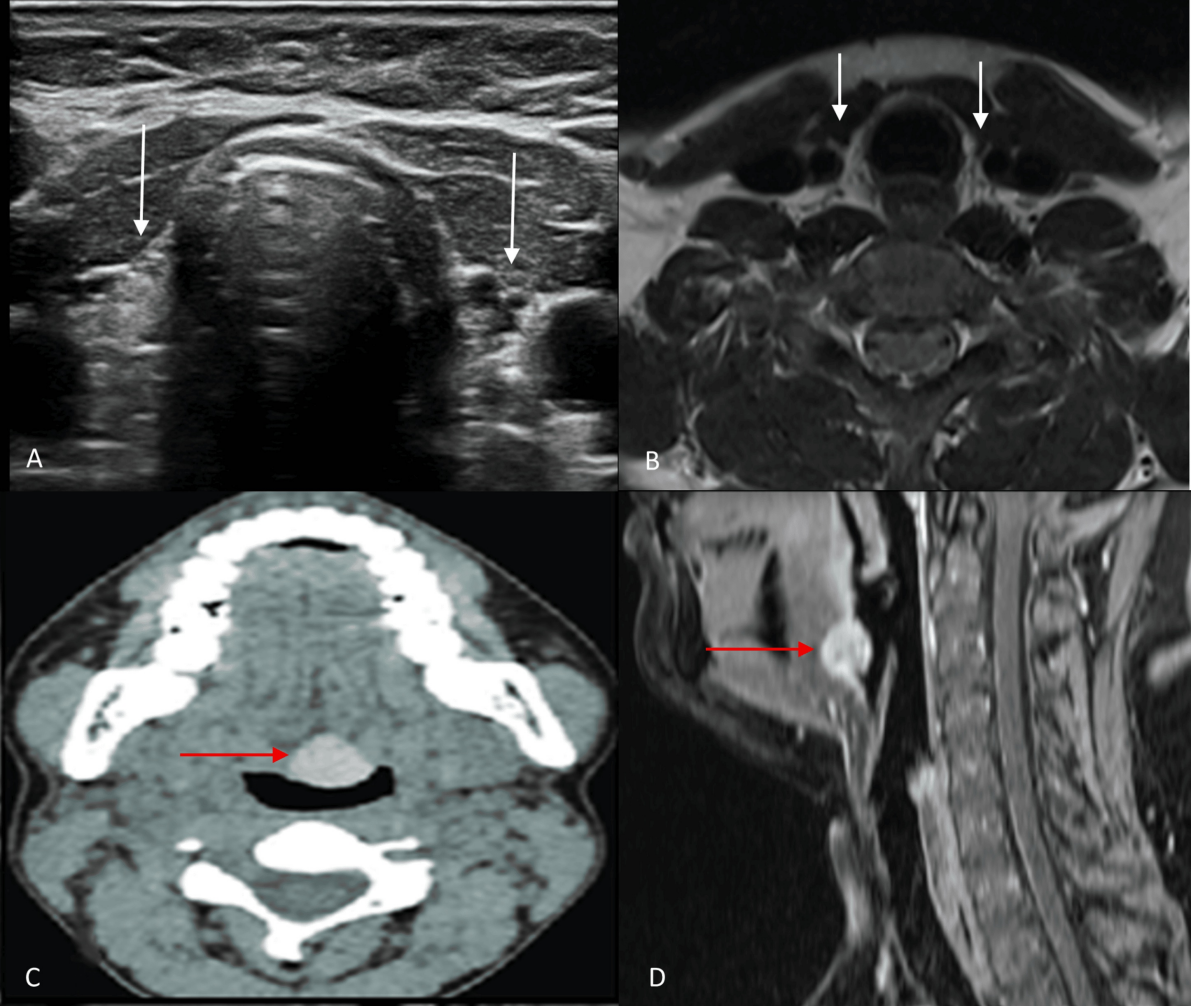
Ultrasound, CT, and MRI images of the thyroid region. (A) Gray-scale ultrasound image and (B) axial T2-weighted MRI of the thyroid region show non-visualization of the thyroid gland in its normal anatomical position (white arrows). (C) Non-contrast CT shows a hyperdense soft tissue mass at the base of the tongue (red arrow). (D) Mid-sagittal section of the post-contrast T1-weighted image shows homogenously enhancing mass at the base of the tongue (red arrow), likely representing lingual thyroid. CT, computed tomography; MRI, magnetic resonance imaging

A non-contrast computed tomography (NCCT) scan revealed another round hyperdense lesion at the base of the tongue, likely lingual thyroid (Figure 3). On contrast-enhanced CT, the rest of the findings described in the ultrasound were confirmed. Additionally, a neck contrast-enhanced magnetic resonance imaging (CE-MRI) scan revealed a well-defined mixed solid cystic lesion in the right submandibular region, directly posterior and inferior to the right submandibular gland. The peripheral solid component showed a T1, T2 isointense signal and heterogeneous contrast enhancement. The central cystic component showed a thick linear enhancing internal septation. Another irregular heterogeneously enhancing lesion was located in the right paramedian infrahyoid region, showing loss of fat planes with the right thyrohyoid and omohyoid muscles. The lesion showed no obvious diffusion restriction. Furthermore, the MRI showed a well-defined mass in the base of the tongue with no clear communication with the other two lesions (Figure 3). Scintigraphy was not performed due to a recent use of an iodinated contrast medium for CECT.

The patient underwent ultrasound-guided fine-needle aspiration cytology (FNAC) for the right submandibular lesion; however, the cytology was non-diagnostic due to the predominant cystic component and rich vascularity. Because of suspicion of malignancy, non-diagnostic cytology, and a recent increase in the size of the swelling, under general anesthesia, the submandibular and infrahyoid lesions were completely surgically resected. Histopathology revealed cystic colloid tissue with benign thyroid cells displaying oncocytic changes and normal thyroid tissue in the submandibular and infrahyoid lesions. The patient was ultimately diagnosed with multifocal benign ectopic thyroid tissue without malignant transformation. The patient was instructed to continue with the medication for hypothyroidism.

## Discussion

The thyroid gland normally begins to develop in utero during the fourth week of gestation. From an embryological perspective, the thyroid gland is formed from two lateral cell clusters and a median cell cluster. It advances to its definitive anatomical location, situated between the second and fifth tracheal cartilages, often by seven weeks of gestation [1].

Ectopic thyroid tissue is the consequence of the failure of the thyroid gland to descend in a normal manner. Ectopic thyroid tissue may be located anywhere along the migration tract of the thyroid gland, from the foramen caecum to the mediastinum. This condition most commonly manifests in the midline cervical region. The lingual thyroid, located at the base of the tongue, accounts for 90% of ectopic thyroid cases. In addition, the thyroid gland can be located in the sublingual or prelaryngeal region, which is at or slightly below the hyoid bone [5,6]. The uncommon occurrence of non-midline ectopic thyroid, also known as lateral aberrant thyroid tissue, can be explained by an interruption in the migration of one of the lateral thyroid anlagen [5,6].

The following are frequently made differential diagnoses for ectopic submandibular thyroid: tumors of the inferior pole of the parotid gland, submandibular inflammatory or malignant lesions, or cervical lymphadenopathy [3]. Thyroid ectopia, however uncommon, should be included in the differential diagnoses of a submandibular mass, which is distinct from the submandibular gland, as demonstrated in our observation. Although ectopic thyroids are often asymptomatic, they might show symptoms when goiters, hyperthyroidism, or malignancy occur. It is necessary to distinguish submandibular ectopic thyroid from metastatic carcinoma of the thyroid. Ectopic thyroids undergo malignant transformation at a pace similar to that of thyroids situated properly [7].

Possible differential diagnoses for a lingual thyroid include metastatic thyroid carcinoma, lymphatic malformations, squamous cell carcinoma at the base of the tongue, dermoid cysts, epidermoid cysts, lingual abscesses, lingual thyroglossal duct cysts, branchial cleft cysts, and dermoid and epidermoid cysts [5-7].

The diagnosis of ectopic thyroid tissue is aided by scintigraphy using technetium (Tc-99m) and iodine in conjunction with ultrasound. All thyroid tissue locations, including hyperfunctional parenchyma, can be detected with thyroid scintigraphy scanning. Additional imaging modalities, such as CT and MRI scans, may be required for loco-regional assessment. The confirmation of an ectopic thyroid diagnosis is often achieved through biopsy or FNAC; however, histologic evaluation may be required in certain instances for the distinction between benign and malignant lesions [8].

Despite their rarity, there are instances of neck masses that were initially suspected to be ectopic thyroid tissue but were subsequently confirmed as metastases of thyroid carcinoma. Additionally, there are instances in which the thyroid ectopia may contain a primary malignant neoplasia [8].

Ectopic thyroid treatment is determined by various parameters, including the size of the mass, local symptoms, the age of the patient, the functional state of the thyroid gland, and potential consequences such as ulceration, hemorrhage, or malignancy. In situations when there is a possibility of malignancy, persistent hyperthyroidism, compressive symptoms, or aesthetic considerations, the surgical excision of the ectopic thyroid gland may be necessary. It is imperative to assess the functionality of additional thyroid tissues prior to surgery to mitigate the risk of iatrogenic hypothyroidism. Furthermore, in up to 70% of patients who have a lateral cervical ectopic thyroid in conjunction with an orthotopic thyroid, the ectopic tissue may be the only functional thyroid [3].

## Conclusions

The presented case highlights the importance of considering ectopic thyroid as a differential diagnosis in submandibular, infrahyoid, and lingual masses, particularly in the absence of an orthotopic gland, although this is rare. The diagnosis of ectopic thyroid tissue is aided by scintigraphy using technetium (Tc-99m) and iodine in conjunction with ultrasound. Biopsies or FNAC are used to confirm the diagnosis of ectopic thyroid. The most suitable therapeutic approach is surgical resection and pathologic evaluation, as these lesions may contain primary carcinoma or metastases of concealed carcinoma of the thyroid.
